# Polymorphisms within inflammatory genes and colorectal cancer

**DOI:** 10.1186/1477-5751-5-15

**Published:** 2006-10-24

**Authors:** Stefano Landi, Federica Gemignani, Fabio Bottari, Lydie Gioia-Patricola, Elisabet Guino, María Cambray, Sebastiano Biondo, Gabriel Capella, Laura Boldrini, Federico Canzian, Victor Moreno

**Affiliations:** 1 Genetics-Department of Biology, University of Pisa, via S. Giuseppe 22, 56126, Pisa, Italy; 2 International Agency for Research on Cancer, 150 Cours Albert Thomas, 69372 Lyon, France; 3 Institut Catala d'Oncologia, Hospitalet, Barcelona, Spain; 4 Laboratori d'Estadistica i Epidemiologia, Facultat de Medicina, Universitat Autonoma de Barcelona, Barcelona, Spain; 5 Department of Surgery, AOUP, via Roma 57, 56126, Pisa, Italy; 6 Unidad de Cirugía Colorrectal, Hospital Universitario de Bellvitge, Hospitalet, Barcelona, Spain; 7 Department of Chirurgic, Area Vasta Nord-Ovest (Toscana), S. Chiara Hospital, Pisa, Italy; 8 Genomic Epidemiology Group, German Cancer Research Center (DKFZ), Im NeuenheimerFeld 580, D-69120 Heidelberg, Germany; 9 IDIBELL, Hospital Universitari de Bellvitge, Hospitalet, Barcelona, Spain

## Abstract

**Background:**

Chronic inflammation is a risk factor for colorectal cancer and polymorphisms in the inflammatory genes could modulate the levels of inflammation. We have investigated ten single nucleotide polymorphisms (SNPs) in the following inflammation-related genes: *TLR4 *(Asp299Gly), *CD14 *(-260 T>C), *MCP1 *(-2518 A>G), *IL12A *(+7506 A>T, +8707 A>G, +9177 T>A, +9508 G>A), *NOS2A *(+524T>C), *TNF *(-857C>T), and *PTGS1 *(V444I) in 377 colorectal (CRC) cancer cases and 326 controls from Barcelona (Spain).

**Results:**

There was no statistically significant association between the SNPs investigated and colorectal cancer risk.

**Conclusion:**

The lack of association may show that the inflammatory genes selected for this study are not involved in the carcinogenic process of colorectum. Alternatively, the negative results may derive from no particular biological effect of the analysed polymorphisms in relation to CRC. Otherwise, the eventual biological effect is so little to go undetected, unless analysing a much larger sample size.

## Background

Epidemiological and biological data show a clear association between chronic inflammatory conditions and subsequent malignant transformation in the inflamed tissue [1]. Inflammatory state is typically accompanied by generation of free radicals, stimulation of cytokines, chemokines, and growth and angiogenic factors that favor tumorigenesis by damaging DNA [2,3], stimulating angiogenesis [4], and by inducing cell proliferation [5,6]. Following these considerations, some pro-inflammatory genes have been shown to be important for the maintenance and progression of cancer [7], including colorectal cancer (CRC) [8,9].

Toll-like and CD14 receptors are examples of pattern recognition receptors that detect antigenic molecules on the surface of gram-positive (peptidoglycans, lipoteichoic acid) and gram-negative (lipopolysaccharide, LPS) bacteria [10]. Polymorphisms of *TLR4 *gene appear to be able to alter inflammatory responses in numerous experimental and clinical models of inflammation. Asp299Gly polymorphism [dbSNP: rs4986790] within *TLR4 *has been investigated by several groups and associated with an increased response to LPS [11]. *CD14 *is a gene preferentially expressed on monocytes/macrophages generating a surface protein. It binds lipopolysaccharide-binding protein and recently has been shown to bind apoptotic cells [10]. The polymorphism -260 T>C [dbSNP: rs2569190] has been shown to increase transcriptional activity by lowering the affinity of the GC box for Sp3, a factor known to inhibit the activity of a number of promoters. This enhanced transcriptional activity has been associated with higher concentrations of soluble CD14 and enhanced CD14 expression on the membrane of the monocytes [12].

MCP-1 is a chemokine that is thought to be responsible for monocyte and T-lymphocytes recruitment in acute inflammatory conditions and may be an important mediator in chronic inflammation. The G allele of -2518 A>G *MCP1 *polymorphism [dbSNP: rs1024611] was found to increase MCP-1 expression [13].

*IL12A *encodes a subunit (alpha) of a cytokine that is required for the T-cell-independent induction of interferon (IFN)-gamma. IL-12 was believed to be unique in its ability to induce the differentiation of native T cells toward the TH1 phenotype and in its pathogenic activity, as shown in various disease models including inflammatory bowel disease (IBD) [14]. SNPs in the regulatory sequence of IL12A are presumed to be associated with the differential production of this cytokine [15].

Focusing on pathways involved more specifically in chronic inflammatory bowel disorders and CRC, the nitric oxide (NO) is a versatile molecule with actions ranging from haemodynamic regulation to anti-proliferative effects on vascular smooth muscle cells [16]. NO is produced by the nitric oxide synthases, endothelial NOS (eNOS), neural NOS (nNOS), and inducible NOS (iNOS). *NOS2A *is the encoding gene for the inducible NOS. It is required for the signalling process in the innate immunity [17] and it plays a central role on chronic inflammatory bowel disorders [18].

Tumor necrosis factor (*TNF*; formerly known as TNFα) and lymphotoxin-α (*LTA*), originally characterized by their ability to induce tumor cell apoptosis and cachexia, are now considered as central mediators of a broad range of biological activities (for a review see [19] and [20]). These activities encompass beneficial effects for the host in inflammation and in protective immune responses against a variety of infectious pathogens [21]. In addition, it has been demonstrated that the core members of the TNF superfamily, including LTα, play an essential role during the organogenesis of secondary lymphoid organs and the maintenance of the architecture of lymphatic tissues [21]. Although a large study did not show association between polymorphisms within LTA-TNF region on chromosome 6 and IBD [22], the role of LTA in the etiology of IBD has been well ascertained in mice [23].

Several lines of evidence suggest that cyclo-oxygenases (COX) 1 and 2 enzymes, encoded by *PTGS1 *and *PTGS2 *genes, play a significant role in colon carcinogenesis. For example, rapid metabolism of arachidonic acid and elevated levels of COX enzymes are found in several cancers, including CRC [24]. In addition, COX enzymes have been shown to stimulate cell proliferation, angiogenesis, and metastasis, and to inhibit apoptosis [25]. During the inflammatory state COX enzymes are elevated, thus the regular use of non-steroidal anti-inflammatory drugs (NSAIDs) could be associated with a reduced risk of CRC, through their inhibition [26]. While COX-2 is rapidly inducible and its expression is usually elevated at sites of inflammation, COX-1 is considered constitutive and is thought to be the main responsible for the cytoprotective production of prostaglandins. However, the traditional description of COX-1 as a purely constitutive, housekeeping gene has been recently challenged by several studies, which have found that COX-1 production can also be regulated at the transcriptional level [27].

In previous investigations, we performed a case-control association study on several polymorphisms in genes involved in the inflammation (namely: *IL6*, *IL8*, *PPARG*, *TNF*, *NFKB1 *and *PTGS2*) and we found associations with the risk of sporadic colorectal cancer [9,28]. In the present study, in order to evaluate whether single nucleotide polymorphisms (SNPs) within more inflammatory genes were associated with the risk to develop CRC, we investigate ten SNPs in the following inflammatory genes: *TLR4 *(Asp299Gly), *CD14 *(-260 T>C), *MCP1 *(-2518 A>G), *IL12A *(+7506 A>T, +8707 A>G, +9177 T>A, +9508 G>A), *NOS2A *(+524T>C), *TNF *(-857C>T), and *PTGS1 *(V444I) in 377 cases and 326 controls.

## Results and Discussion

Each polymorphism was in Hardy-Weinberg equilibrium in controls. The main effects related to each polymorphism by case/control status are shown in Table 1. The odd ratios (ORs) and 95% confidence interval (CI) are shown for the codominant model. For the variant V444I within *PTGS1*, we found an allele frequency too low to draw any conclusion, and a much larger study is necessary to characterize better whether this SNP could play any role in the risk of CRC. All the other polymorphisms investigated showed clearly a lack of statistically significant association with CRC. The dominant or log-additive models did not change substantially the results (data not shown). No association was found when cases were classified according to cancer site (rectal, left colon or right colon, data not shown). The analyses for association based on interactions of these polymorphisms with dietary variables (alcohol, tobacco, coffee, vegetables, meat and meat products, fats, fruits, BMI, calories) did not elicit any statistical association (data not shown).

**Table 1:** Odd ratios and 95% confidence intervals (95% CI) for colorectal cancer by genotypes under investigation.

**Gene and SNP**	**Controls N°**	**Cases N°**	**OR (95%CI)**	***P*_*codominant*_^1^**	***P*_*additive*_^2^**
*TLR4 *Asp299Gly					
AA	232	251	1		
AG	37	31	0.75(0.45–1.27)	0.29	0.29
GG	0	0	N.A.		
*CD14 *-260 T>C					
TT	63	68	1		
TC	137	151	0.98(0.65–1.50)	0.82	0.58
CC	65	62	0.87(0.53–1.43)		
*MCP1 -2518 A>G*					
AA	138	161	1		
AG	97	97	0.86(0.6–1.24)	0.71	0.46
GG	16	18	0.88(0.43–1.82)		
*IL12A +7506 A>T*					
AA	97	102	1		
AT	108	136	1.19(0.81–1.74)	0.49	0.99
TT	41	37	0.90(0.53–1.53)		
*IL12A +8707 A>G*					
AA	75	69	1		
AG	121	149	1.30(0.86–1.96)	0.25	0.87
GG	68	59	0.94(0.58–1.53)		
*IL12A +9177 T>A*					
TT	100	108	1		
TA	121	140	1.04(0.72–1.51)	0.73	0.68
AA	43	39	0.85(0.51–1.43)		
*IL12A +9508 G>A*					
GG	104	106	1		
GA	124	135	1.05(0.73–1.52)	0.85	0.85
AA	40	36	0.91(0.53–1.54)		
*NOS2A +524T>C*					
TT	93	92	1		
CT	127	142	1.15(0.79–1.68)	0.76	0.56
CC	45	49	1.12(0.68–1.86)		
*TNF -857C>T*					
GG	220	219	1		
GA	45	58	1.32(0.85–2.04)	0.41	0.19
AA	3	4	1.55(0.34–7.09)		
*PTGS1 V444I*					
GG	265	280	1		
GA	5	3	0.52(0.12–2.22)	0.36	0.36
AA	0	0	N.A.		

^1 ^P-value for the codominant model, comparing heterogeneity among three genotypes

^2 ^P-value for the log-additive model, comparing the effect of each additional variant allele

The lack of association can be due to three main reasons. First. In spite of the importance of the inflammation for the carcinogenic process of the colorectum, we, unfortunately, selected some of the inflammatory genes that do not have a role in this process. Second. It is also possible that, indeed, the genes have a role for CRC, however the selected polymorphisms do not have biological effects for CRC. Third, it is possible that some of the SNPs have a small effect, which undergo undetected unless studied by analysing a much larger sample size. Although the present study does not seem to support a role for inflammatory polymorphisms, it should be stressed that our previous work did show a role for a polymorphism within the promoter of *IL6 *and the polymorphism at codon 12 of *PPARG *[9]. However, *IL6 *and *PPARG *may be considered also as part of alternative pathways not directly linked to inflammation. *IL6 *is involved in the cross-talk between neutrophiles passed into the colon lumen and colocytes [9], whereas *PPARG *is involved in the resistance to insuline [9], thus, overall, our results seems to point towards other mechanisms, rather than stressing the role of inflammation for the CRC.

## Conclusion

According to the results of our study, we conclude that the SNPs analysed do not have a strong impact on the risk of CRC.

## Methods

Cases (n = 377) were patients with a new diagnosis of CRC attending a University Hospital in Barcelona, Spain, between January 1996 and December 1998. All cases have had histological confirmation of their tumor diagnosis. Participation rate among cases was 72%. Cases who did not participate in the study were similar to those included with respect to age, sex, tumor location, and extent.

Controls (n = 326) were randomly selected among patients admitted to the same hospital during the same period. To avoid selection bias, only incident cases were selected. Participation rate among controls was 69.4%. All subjects had the same ethnicity (Caucasian). The ethical committee of the hospital approved the study and subjects gave informed consent at recruitment. Interviews of cases and controls were performed by trained personnel, using a structured test to determine demographic characteristics and potential risk factors for CRC. A more detailed report on study design has been previously published [9]. SNPs were selected based on previous publications from the literature. An extensive literature research was carried out with the aim to study the potential role of some inflammatory SNPs for CRC, once the information was available for other studies.

Genotyping was carried out with the 5' nuclease assay (TaqMan) by minor groove binder (MGB) probes fluorescently labelled with FAM or VIC and using the protocol recommended by the supplier (Applied Biosystems, Foster City, CA). To ensure quality control, DNA samples from cases and controls were randomly distributed on PCR plates, and all genotyping was conducted by personnel who were blinded to case – control status. Only genotype calls scored concordantly by two independent trained operators were retained. Finally, a random 8% of the samples were re-genotyped blindly. The probes and primers for the genotyping reactions are reported in Table 2. More details on the genotyping technique were reported previously [9].

**Table 2:** PCR primer and TaqMan^® ^Probe Sequences

Gene	Rs number	Trivial name	Primer Forward	Primer Reverse	VIC Probe	6FAM Probe
*TLR4*	rs4986790	Asp299Gly	CCATTGAAGAATTCCGATTAGCATA	CACTCACCAGGGAAAATGAAGAA	CCTCGATGATATTATT	CTCGATGGTATTATTG
*CD14*	rs2569190	-260 C>T	GGAAATATTGCAATGAAGGATGTTT	CTAGATGCCCTGCAGAATCCTT	CTGTTACGGTCCCC	TGTTACGGCCCCC
*MCP1*	rs1024611	-2518 A>G	GGGAGGGCATCTTTTCTTGAC	GGTGAAGGGTATGAATCAGAAAAGA	ACAGCTATCACTTTC	AGACAGCTGTCACTTT
*IL12A*	rs2243138	+7506 A>T	AACCAGGAGTCCCCGATCC	GGCCCACTGCCCAACAG	CCATGGAGTGGTACTG	AGGCCATGGTGTGGT
*IL12A*	rs668998	+8707 A>G	CCAATCTTTCTCCCTGTAAATGTGTT	TGAATGTCTAATAGGGCAAGAATTTG	TGCATGACAAACAT	CTGCATGGCAAACA
*IL12A*	rs2243151	+9177 A>T	TTATTGCATGGTTAGTTTTTCACTTTTT	GGAGCATTGGAATGATTTGGTT	AGCATACTACACTACTTG	AGCATACTACACAACTT
*IL12A*	rs2133310	+9508 G>A	GGGACCTAATTAACTGTGTTATTGTGA	AGCGGGTGTTCTGATGTCTTG	AGTGCCAACACCTAG	TGCCAGCACCTAGT
*NOS2A*	rs944722	+524G>A	CTCTGTTTCTCTGATCCCACTTTCT	GTGATATGAGATCTCGCCACTACACT	TGGAGTCTCTGTCACC	ATGGAGTTTCTGTCACC
*TNF*	rs1799724	-857C>T	GGTAGGAGAATGTCCAGGGCTAT	AGGTCCTGGAGGCTCTTTCACT	CCCTGTCTTCGTTAAG	CCCTGTCTTCATTAAG
*PTGS1*	rs5794	V444I	GCATGAAACCCTACACCTCCTT	GGGACTGCATCCAGGAAACA	AGGAGCTCGTAGGTGA	CAGGAGCTCATAGGT

Each polymorphism was tested in controls to ensure that it was in Hardy-Weinberg equilibrium. To test the hypothesis of association between genetic polymorphisms and CRC, multivariate methods based on logistic regression analyses were used. When cases were subdivided into groups, polytomous logistic regression was used, comparing each group of cases with the whole set of controls. Odds ratios (OR) and 95% confidence intervals (CI) were calculated for each group compared to the homozygotes for the most common allele (set as having risk = 1). For polymorphisms, homozygosity for the more frequent allele among controls was set as the reference class. Analyses were initially performed under a co-dominant model (three genotypes separated). A log-additive model to assess the effect of each additional rare allele and a codominant model were also explored to increase the statistical power. All analyses were adjusted for age and sex and p values were derived from likelihood ratio tests. More details concerning the statistical analyses were published previously [9].

The sample size of our study, for a rare allele frequency of 5%, is enough to detect ORs greater than 2 with more than 90% power assuming a log-additive model. For allele frequencies of 15%, the power to detect an OR of 1.5 is 82%. For allele frequencies of 35% as observed for some SNPs in this study, the power to detect an OR of 1.3 is 66%.

## Competing interests

The author(s) declare that they have no competing interests.

## Authors' contributions

SL, FB, LB, FC, VM contributed to the draft of the manuscript. FG, LGP, SL contributed to the wet lab. SL carried out the database and literature search for the selection of the candidate genes and polymorphisms. EG contributed to the statistical analysis. MC, SB contributed in the recruitment and follow-up of patients. GC and VM designed the case-control study. FC run the laboratory. All authors read and approved the final manuscript.
